# Shortcomings of Trials Assessing Antidepressants in the Management of Irritable Bowel Syndrome: A Critical Review

**DOI:** 10.3390/jcm9092933

**Published:** 2020-09-11

**Authors:** Sun Jung Oh, Will Takakura, Ali Rezaie

**Affiliations:** 1 Johns Hopkins Hospital, Baltimore, MD 21287, USA; soh51@jhmi.edu; 2 Cedars-Sinai Medical Center, Los Angeles, CA 90048, USA; Will.Takakura@cshs.org

**Keywords:** antidepressive agents, tricyclic antidepressants, irritable bowel syndrome, publication bias, healthcare quality assessment, serotonin reuptake inhibitors

## Abstract

Irritable bowel syndrome (IBS) is a common disorder requiring complex, multidisciplinary management. Antidepressants are commonly used and recommended in guidelines for the treatment of patients with IBS. We assessed randomized controlled trials (RCTs) on antidepressants in patients with IBS, with specific attention to study design and data quality/reporting characteristics. Following a comprehensive search, data and RCT characteristics were systematically summarized. Fragility index, representing the number of positive “events” that the study relies on for its significance, was calculated. Eighteen RCTs were included. Overall, tricyclic antidepressants (TCAs), but not selective serotonin reuptake inhibitors (SSRIs), appeared to be efficacious in IBS. Eight studies reported on adverse events (AEs), which were significantly greater in patients receiving antidepressants versus placebo. The median (mean) fragility index of TCA trials was 0 (1.5). RCTs with positive results had significantly lower placebo rates (20.8%) versus negative studies (45.7%; *p* < 0.0001). RCTs exhibited limitations related to study design (sample size and blinding), data analysis (outcomes and placebo response), and data reporting (selective reporting of AEs and publication bias). Careful consideration of limitations of RCTs on antidepressants in IBS is warranted to formulate a safe and beneficial treatment regimen for patients with IBS.

## 1. Introduction

Irritable bowel syndrome (IBS) is characterized by recurrent abdominal pain associated with defecation and with alterations in stool frequency or form [1]. Patients affected by IBS also commonly experience bloating and abdominal distention. It is a common, chronic condition estimated to affect 11.2% of individuals worldwide [2]. IBS is further classified by the predominant stool form observed during >25% of bowel movements: IBS with constipation (IBS-C), IBS with diarrhea (IBS-D), and IBS with mixed bowel habits [1]. Patients with IBS often have impairments in quality of life [3,4]. Indeed, the severity of IBS symptoms was shown to be associated with daily activity impairment [4]. The negative effects of IBS also extend into patients’ professional lives. In a study of patients with IBS, 24.3% of employed patients had been absent from work during the previous week, and 86.8% had experienced a decrease in work productivity [4].

Patients with IBS often experience psychological comorbidities, such as anxiety and depression. A meta-analysis of 27 studies showed levels of anxiety (pooled standardized mean difference [SMD], 0.84; 95% confidence interval (CI), 0.67–1.01; *p* < 0.001) and depression (pooled SMD, 0.76; 95% CI, 0.62–0.90; *p* < 0.001) were significantly higher in adults with IBS compared with healthy controls [5]. However, it is unclear whether the high rate of depression among patients with IBS is part of the disease pathophysiology or a direct result of the chronic, relapsing, and debilitating nature of IBS [6]. Nevertheless, antidepressants have been used for >40 years and are recommended in societal guidelines for treatment of IBS patients with or without concomitant psychiatric disorders. The American College of Gastroenterology recommends tricyclic antidepressants (TCAs) for overall symptom improvement in IBS patients (recommendation: strong; quality of evidence: high) and also suggests selective serotonin reuptake inhibitors (SSRIs; recommendation: weak; quality of evidence: low) [7]. In contrast, American Gastroenterological Association guidelines give a conditional recommendation for TCAs (low quality of evidence) but conditionally recommend against SSRIs for IBS (low quality of evidence) [8]. Notably, none of the eight drugs that have received US Food and Drug Administration (FDA) approval for IBS (alosetron, eluxadoline, linaclotide, lubiprostone, rifaximin, plecanatide, tegaserod, and tenapanor) or European Medicines Agency (EMA)-approved drugs (eluxadoline and linaclotide) have antidepressant properties [9]. Low-grade inflammation, bile acid malabsorption, dysmotility, and gut microbiome dysbiosis have important roles in the etiology of IBS [7,10,11]. However, antidepressants have little modulatory effect on these factors [7].

Several meta-analyses and systematic reviews have been performed on the use of antidepressants in IBS, with the main focus on efficacy rather than the side-effect profile and trial data quality [12,13,14]. The current aim was to critically review the efficacy of antidepressants in IBS reported in clinical trials and comprehensively review the side-effect profile, quality of evidence, and strengths and weaknesses of the identified trials.

## 2. Methods

Relevant clinical studies and systematic reviews were identified by searching the PubMed and EMBASE databases from 1966 to 30 September 2019, for articles in any language, using the following search terms: “antidepressants,” “tricyclic antidepressant,” “selective serotonin reuptake inhibitor,” “irritable bowel syndrome,” “depression,” “anxiety,” and “comorbid.” The risk of bias was assessed based on guidelines provided in the Cochrane Handbook for Systematic Reviews of Interventions [15] by recording the method used to generate the randomization schedule and conceal treatment allocation; whether blinding was implemented for participants, personnel, and outcomes assessment; what proportion of patients completed follow-up; whether an intention-to-treat analysis was extractable; and whether there was evidence of selective reporting of outcomes. Two investigators performed this assessment independently, and disagreements were resolved by consensus. Fragility index was calculated by converting the number of patients who were considered “non-responders” to “responders” in order for the *p*-value to become > 0.05 using the Fisher’s exact test.

## 3. Efficacy of Antidepressants in IBS

Twelve randomized controlled trials (RCTs) of TCAs and seven RCTs of SSRIs for IBS (Table 1) are commonly cited in meta-analyses and systematic reviews (referenced in ≥20 articles, based on literature search [12,13,14,16,17,18,19,20,21,22,23,24,25,26,27,28,29,30,31,32]), and these 18 trials (one trial evaluated both a TCA and an SSRI) were published between 1978 and 2017 [33,34,35,36,37,38,39,40,41,42,43,44,45,46,47,48,49,50].

**Figure 1:**
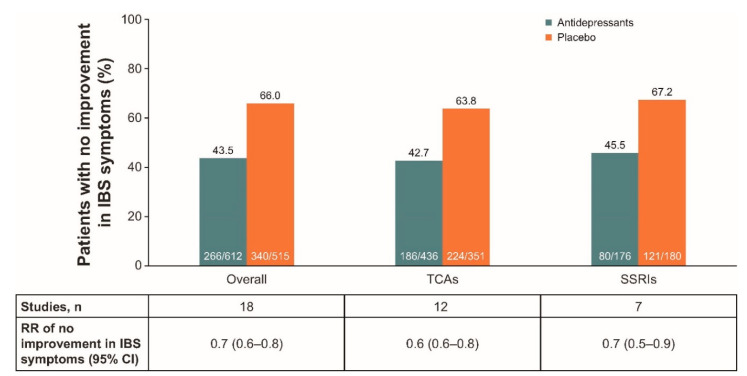
Efficacy of antidepressants for the treatment of IBS [12]. CI: confidence interval, IBS: irritable bowel syndrome, RR: relative risk, SSRI: selective serotonin reuptake inhibitor, TCA: tricyclic antidepressant.

Antidepressants used to treat patients with IBS include TCAs and SSRIs [51]. In a 2019 meta-analysis of data from 18 RCTs in patients with IBS, results showed that patients taking antidepressant therapy reported a lower percentage of no improvement compared with those taking placebo, and results were similar when patients were subcategorized as taking either TCAs or SSRIs (Figure 1) [12].

In a meta-analysis of 12 RCTs (TCAs (*n* = 5), SSRIs (*n* = 6), TCAs and SSRIs (*n* = 1)) published in 2015, evaluation of nine studies reporting global symptom relief showed that antidepressants improved global symptoms of IBS (relative risk (RR), 1.4, 95% confidence interval (CI), 1.1–1.8) [18]. In a subgroup analysis, TCAs had a beneficial effect on improving global symptoms of IBS (*n* = 5 studies; RR, 1.4, 95% CI, 1.1–1.7), whereas treatment with SSRIs did not have a significant effect on global symptoms (*n* = 5 studies; RR, 1.4, 95% CI, 0.8–2.3) [18]. However, the five SSRI studies included three different medications (paroxetine, citalopram, and fluoxetine). In an analysis of abdominal pain, data from three studies (TCAs (*n* = 1), SSRIs (*n* = 3)) indicated that antidepressants did not significantly improve this symptom (mean difference, −8.9, 95% CI, −19.7 to 2.0) [18].

## 4. Adverse Events with Antidepressant Therapy

A 2019 systematic review and meta-analysis acknowledged that adverse events (AEs) were poorly reported in trials of antidepressants in patients with IBS [12]. A pooled analysis of data from the eight studies that reported AEs showed the incidence of AEs was significantly greater in patients treated with antidepressants than in those who received placebo (36.4% (83 out of 228) vs. 21.1% (47 out of 223); RR, 1.6, 95% CI, 1.2–2.0), with a number needed to harm of 8.5 (95% CI, 5–21) [12]. No serious AEs were reported. Of the eight studies that used TCAs, six showed that a significantly greater number of AEs occurred with TCAs compared with placebo (RR, 1.6; 95% CI, 1.2–2.1) [12]. However, AE data were incompletely reported by many of the RCTs.

Among ten individual trials of antidepressants for the treatment of IBS that reported data for TCAs, five reported higher discontinuation rates with a TCA versus placebo, with the highest percentage of discontinuations associated with administration of imipramine (Table 1) [33,35,37,38,40]. Similarly, discontinuations due to AEs were greater among patients treated with TCAs compared with placebo (Table 1) [33,36,37,38,40]. Although not consistently reported, commonly reported AEs of TCAs have been drowsiness (range, 16–24%), dry mouth (12–48%), and fatigue (6–15%) [33,34,37,40,41].

In a meta-analysis of 12 RCTs, two studies examined the occurrence of individual AEs with SSRIs; in these two studies the pooled RR of patients experiencing headache, poor sleep, anxiety, and nausea were 0.8 (95% CI, 0.3–2.2), 1.0 (95% CI, 0.4–2.5), 2.0 (95% CI, 0.5–7.6), and 1.0 (95% CI, 0.4–3.0), respectively [18]. For SSRIs, the percentages of patients experiencing individual AEs were reported in two studies [45,50]. In a study of 72 patients receiving paroxetine or placebo, the most commonly reported AEs were drowsiness (36.1% vs. 25.0%, respectively), dry mouth (27.7% vs. 16.6%), sexual dysfunction (female, 25.8% vs. 12.5%; male, 20.0% vs. 0%), poor sleep (16.6% vs. 13.8%), and nightmares/vivid dreams (16.6% vs. 13.8%) [50]. In a study of 44 patients receiving fluoxetine or placebo, the most common AEs in the fluoxetine group were anorexia (22.7% vs. 4.5%, respectively) and headache (22.7% vs. 18.2%) [45].

## 5. Methodologic Considerations for Evaluating Antidepressant Data in IBS Clinical Studies

### 5.1. Sample Size

**Figure 2:**
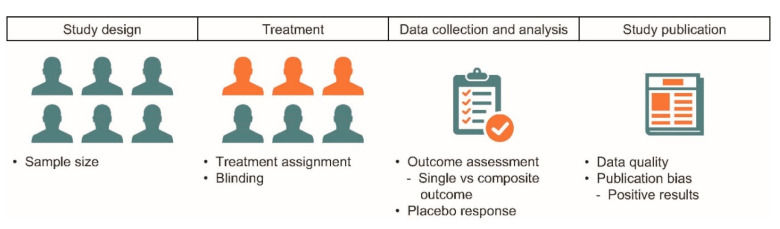
Methodologic characteristics assessed for clinical studies of antidepressant treatment in patients with irritable bowel syndrome.

Multiple methodologic characteristics of clinical trials of antidepressant therapies for IBS were systematically assessed (Figure 2). 

The pooled mean number of patients randomly assigned to any antidepressant treatment arm in Table 1 was 35 (range, 11–144; median, 27), indicating a small sample size across the various published studies [33,34,35,36,37,38,39,40,41,42,43,44,45,46,47,48,49,50]. Inadequate power in clinical studies of antidepressants, due to insufficient sample size, may overestimate the desired effects of antidepressants relative to a control therapy [52]. A 2013 meta-epidemiologic study showed that when RCTs were stratified by sample size, treatment effect estimates were significantly larger in smaller trials than in the largest trials [53]. As the sample size increases, the variance in the results (as well as the placebo effect) converges. Thus, results from smaller studies have larger CIs and overestimate the treatment effect, so they should be interpreted with caution [53].

### 5.2. Pooling of Data on Different Classes of Antidepressants

There are various classes of antidepressants, and even drugs within the same class have dramatic differences in efficacy and AE profiles. This analysis included six antidepressants from the TCA class (desipramine, trimipramine, amitriptyline, doxepin, imipramine, and nortriptyline) and three from the SSRI class (fluoxetine, paroxetine, and citalopram; Table 1). The validity of pooling data for different antidepressants may be similar to pooling data from various biologics. For example, it would not be appropriate to pool data within or across the various classes of biologic agents to obtain a clinically relevant point estimate of the efficacy of biologic therapy in inflammatory bowel disease. A similar situation applies to antidepressants in IBS because pooling data from various classes, or data for drugs within the same class that have significantly different effects, does not necessarily provide a clear picture for healthcare providers or patients.

### 5.3. Blinding

Antidepressants used in IBS trials were already available on the market; hence, patient consent forms are required to clearly state the known AEs of the medication(s) being studied. These disclosures educate the participants about potential AEs of the drug and compromise blinding when the participant experiences such AEs. As a potential solution for this issue, Greenberg and Fisher [54] suggested the use of an “active placebo” such as atropine, which would exhibit some of the same AEs as TCAs. However, such an intervention has never been implemented in trials assessing patients with IBS. In addition, placebo tablets may have a different color, taste, and size if not matched exactly to the trial drug, which may lead to inadvertent unblinding, and care should be taken in selecting excipient compounds that are inert [55].

The most common TCA medications studied for IBS are amitriptyline, desipramine, doxepin, and nortriptyline. These medications have a generally similar AE profile that is well known to healthcare providers and the public and includes dry mouth, drowsiness, dizziness, fatigue, blurred vision, tinnitus, constipation, weight changes, increased perspiration, alterations in libido, and trouble urinating. Among the SSRIs, the most commonly studied medications are citalopram, fluoxetine, and paroxetine, with known AEs of dry mouth, fatigue/somnolence, and difficulty concentrating. Because these are common and well-known AEs, it remains questionable whether patients or investigators are truly blinded to which patients are receiving study drug or placebo in RCTs. These AEs typically do not occur to the same extent in patients receiving placebo, thus increasing the potential for bias [54]. The limitations of blinding were confirmed in a study in which a blinded evaluator, when provided AE profiles from a clinical trial (of etoperidone, a trazodone-like prospective antidepressant), correctly guessed treatment assignment for 72.7% of 22 patients receiving an antidepressant and 66.7% of 12 patients receiving placebo [56]. Similarly, if patients believe they are receiving active drug as part of a double-blind study, this knowledge theoretically will affect success rates [54]. In trials evaluating antidepressants, there was a substantial imbalance of AEs, with a predominance in the trial drugs versus placebo (36.4% [83/228] vs. 21.1% [47/223], respectively) [12], which may have predisposed the studies to unblinding. When evaluating individual studies for the quality of blinding of participants, nine studies were deemed to be high risk due to a larger percentage of patients experiencing AEs in the antidepressant groups (Table 2).

### 5.4. Overall Risk of Bias

As outlined above, two authors (S.J.O. and W.T.) independently evaluated the risk for bias using the Cochrane Risk of Bias Tool for Randomized Controlled Trials, with any disagreements settled by discussion and achieving consensus of all three authors. Studies judged to be high risk in two or more categories were deemed to be poor quality, studies with only one category of high risk were deemed to be fair quality, and those without high risk in any category were deemed to be good quality. Eight of the 12 TCA trials were found to be of poor quality, and one of the seven SSRI trials was shown to be of good quality (Table 2) [33,34,35,36,37,38,39,40,41,42,43,44,45,46,47,48,49,50].

The relatively poor quality of the RCTs of antidepressants in patients with IBS further underscores the need for healthcare providers, patients, and authors of treatment guidelines for IBS to assess the results of these trials and strengthen treatment recommendations to use caution with these agents.

### 5.5. Placebo Rate 

The overall placebo response also has been documented in clinical studies of IBS. A meta-analysis of 73 RCTs in IBS (*n* = 8364) reported a pooled placebo response rate of 37.5% [57]. Our analysis of the 18 studies summarized in Table 1 showed that the overall placebo response rate in the positive antidepressant studies (20.8%) was less than half of that in the negative studies (45.7%; *p* < 0.0001). The 20.8% placebo response rate in positive antidepressant studies is unusually low, compared with the 37.5% placebo response rate reported for all IBS studies [57]. The difference in placebo response rates may be secondary to the blinding bias risk mentioned previously and may play an important role in the significance of the positive studies. In addition, as alluded to by Tack et al., patient characteristics such as the prevalence of psychiatric comorbidities as well as the duration and stability of IBS symptoms may have played a role in varying the placebo rates [46]. In the meta-analysis of 73 RCTs, other factors that increased the placebo effect included reporting of study outcomes by the healthcare provider as opposed to patient reporting (*p* = 0.005) and shorter trial duration (i.e., 1–4 weeks vs. >8 weeks; *p* = 0.004) [57]. All of these flaws could potentially be minimized by designing a large, multicenter trial with common inclusion and exclusion criteria. 

### 5.6. Outcome Assessment

Generally, the primary endpoint in IBS trials is improvement in gastrointestinal (GI) symptoms, usually assessed by a composite score. Use of composite scores may create problems with the interpretation of clinical trials [52], especially given the heterogeneity in GI symptoms associated with IBS [1] or in any other condition in which symptomology may vary among patients [58,59]. Comparison of composite outcome scores as valid clinical endpoints has been debated given that the actual individual symptoms may vary in patients with the same composite score [59]. For example, a Hamilton Depression Rating Scale score of 8 can result from a score of 4 for depressed mood and 4 for feelings of guilt, or from a score of 2 for insomnia, 4 for work and interest, and 2 for anxiety. Thus, the composite score may be identical for a group of patients, but the actual difference in individual symptoms and severity may be better (or worse) for some individuals.

### 5.7. Publication Bias

Clinical studies of antidepressant therapies in IBS reveal evidence of publication bias along with significant heterogeneity between studies (*I*^2^ = 69%, *p* < 0.001) and significant funnel plot asymmetry [12]. The issue of publication bias among antidepressant clinical trials has been subject to a prolonged debate. Turner and colleagues [60] examined reviews from the FDA for studies of antidepressant agents; they found that approximately one-third (31%) of the 74 registered studies had not been published. They also found that whether a study was published was strongly predicted by the outcome: 37 studies with positive results were published, whereas only one positive study was not published. In contrast, of the studies that were viewed as having negative results, only three studies were published, 22 studies were not published, and 11 studies that were viewed by the FDA as having a negative outcome conveyed a positive outcome in the published literature. These data may explain the discrepancy between the published literature showing 94% positive results for the antidepressant trials conducted versus the FDA analysis showing 51% positivity [60]. Further, de Vries and associates [61] reported that 41.1% of studies describing negative results were published as part of pooled analyses, effectively “hiding” them, while 96.3% of studies with positive results were published as independent articles.

It should be noted that, during the FDA regulatory approval process, pharmaceutical companies are obligated to report available data to authorities, regardless of the publication status of a particular agent for a proposed indication. The same situation does not apply when agents are being studied for other disease states without plans for seeking marketing approval in the potential new treatment area (e.g., antidepressants in IBS). Hence, the potential for publication bias in clinical studies of antidepressants [60] for use with conditions other than depression (such as IBS) may be far greater. However, as some of these trials may not be registered, the extent of publication bias may not be fully determined.

### 5.8. Fragility Index

In the age of frequentist analysis with a standardized threshold *p* value of 0.05, the fragility index has been used to assess the strength of RCTs [62]. The fragility index is defined as the number of “non-events” that would need to change to “events” for the *p* value to become ≥ 0.05 using Fisher’s exact test [62,63]. This number represents the number of events the trial relies on for statistical significance; thus, a higher index signifies a stronger study. When considering the RCT data for antidepressants in IBS [23,33,34,35,36,37,38,39,40,41,42,43,44,45,46,47,48,49,50], the calculation of fragility index values showed that eight (66.7%) of the 12 TCA studies and three (42.9%) of the seven SSRI studies had a fragility index of 0 (Table 3).

The maximum fragility index was 6 for both TCA and SSRI studies, and the median (mean) fragility index of TCA and SSRI trials was 0 (1.5) and 1 (1.4), respectively. These results show that, on average, individual studies relied on only a small number of events for significance to be achieved. A high fragility index is moderately correlated with larger studies [62], and thus larger trials are needed to provide stronger evidence for the effectiveness of antidepressants in IBS.

### 5.9. Primary Outcome of Interest

Studies of IBS published before the 2012 guidance from the FDA regarding trial study design and outcomes for assessing the efficacy of agents for the treatment of IBS-D or IBS-C [64] used a variety of composite endpoints, such as patient-reported improvement in global IBS symptoms [12,18]. For example, “Subject Global Assessment of Relief,” a primary endpoint widely used in many trials to assess efficacy, captures patients’ subjective overall well-being based on symptoms of abdominal pain/discomfort and altered bowel habits, using a Likert scale based on the question, “Compared to the way you usually felt before entering the trial, how would you rate your relief of symptoms during the past week?” [64]. Given the high degree of subjectivity of current patient-reported outcomes, the FDA recommends against their use as a single primary outcome measure in IBS trials. Currently, the FDA recommends a composite endpoint that considers responders to be those patients achieving improvements in both abdominal pain intensity and stool consistency [64], which was only used in a few of the IBS trials that assessed antidepressants (Table 1).

Further, studies included in meta-analyses differ in patient populations and the specific therapy used, leading to heterogeneity in the data [12,18]. In the 18 studies summarized in Table 1, Rome II criteria was used most often (38.9%; seven out of 18), followed by no specific criteria (33.3%; six out of 18), Rome I (22.2%; four out of 18), and Rome III (5.6%; one out of 18). IBS subtype majority was not listed in 55.6% (10 out of 18) of studies, 38.9% (seven out of 18) evaluated mainly IBS-D patients, and 5.6% (one out of 18) evaluated IBS-C patients. This heterogeneity makes comparisons across agents difficult and limits any global conclusions that can be drawn.

## 6. Future Directions

While none of the currently available FDA-approved drugs for IBS (alosetron, eluxadoline, linaclotide, lubiprostone, rifaximin, plecanatide, tegaserod, and tenapanor) and European Medicines Agency (EMA)-approved durgs for IBS (linaclotide and eluxadoline) possess antidepressant-like properties, GI societal guidelines support the use of antidepressants in the management of IBS, based on the available literature. All FDA and EMA-approved treatments for IBS have a robust sample size, with a mean sample size of 623 for alosetron, 1078 for eluxadoline, 717 for linaclotide, 455 for lubiprostone, 407 for rifaximin, 871 for plecanatide, 651 for tegaserod, and 629 for tenapanor [7,65,66], whereas trials on antidepressants on average have a sample size of 63 [12]. A small sample size inadvertently leads to a variability in placebo rates, which may suggest a heterogeneity in the study populations between studies. Thus, it is difficult to make the generalizations needed to form a strong recommendation for a global audience for a very common disease such as IBS. In addition, most FDA-approved drugs were tested against FDA’s rigorous composite endpoint, so future studies evaluating antidepressants should have a larger sample size and use the FDA’s endpoint for efficacy. To preserve blinding in the setting of known side effects of antidepressants, the control arm of the studies should receive an active drug with a similar side-effect profile, such as anticholinergics or antispasmodics.

## 7. Conclusions

Clinical studies of antidepressants in IBS are limited by issues related to study design (e.g., sample size, blinding), data analysis (e.g., outcomes, placebo response), and data quality (e.g., selective reporting of AE profile, publication bias). Although studies support the efficacy of antidepressants in IBS, careful consideration of the limitations associated with clinical study designs with antidepressants, and thus their use, is warranted. 

## Figures and Tables

**Table 1:** Summary of clinical studies of antidepressants in patients with irritable bowel syndrome ^a^.

Study Design and Patient Population	Treatment(s)	Key Endpoint ^b^	AEs Reported	Discontinuations
TCAs				
Heefner JD, et al. [38]	Desipramine (*n* = 22) 150 mg/d for 2 mo PBO (*n* = 22)	Percentage of patients with self-reported improvement in abdominal pain or discomfort at 8 wk: ITT (*n* = 31): 85.7% (12/14) desipramine vs. 58.8% (10/17) PBO, *p* > 0.05	Yes	Desipramine: *n* = 8 (36.4%); 3 due to AEs PBO: *n* = 5 (22.7%); 1 due to AEs
Myren J, et al. [39]	Trimipramine (*n* = 30) 25 mg/d for 28 d PBO (*n* = 31)	Improvement from baseline in patient-graded symptom scores at 4 wk: ITT (*n* = 61): ~50% improvement from baseline in individual symptom scores in trimipramine and PBO groups (significant in trimipramine group vs. PBO for vomiting, sleeplessness, and depression)	No	NR
Nigam P, et al. [41]	8 combinations of 3 simultaneous treatments (*n* = 21 per group): A: amitriptyline 12.5 mg/d + 5 mg chlordiazepoxide a: dummy B: hyoscine butylbromide b: dummy C: ispaghula husk c: dummy	Improvement in IBS symptoms at 12 wk (*n* = 168 [21 blocks of 8 patients): 51.2% with any “A” (amitriptyline) combination vs. 23.8% any “a” (dummy) combination; *p* < 0.01	Yes	NR
Boerner D. [44]	Doxepin (*n* = 42) PBO (*n* = 41)	Mean improvement (SD) from baseline in abdominal pain at 8 wk: –0.7 (0.9) with doxepin (*n* = 40) vs. –0.4 (1.0) PBO (*n* = 39; *p* < 0.05)	Yes	Doxepin: *n* = 2 (4.8%) PBO: *n* = 2 (4.9%)
Bergmann ML, et al. [43]	Trimipramine (*n* = 19) PBO (*n* = 16)	Global improvement at 12 wk: trimipramine 73.7% (14/19) vs. PBO 12.5% (2/16); *p* = NR	No	Trimipramine: *n* = 1 (5.3%) PBO: *n* = 3 (18.8%)
Vij JG, et al. [40]	Doxepin (*n* = 25) 75 mg/d for 6 wk PBO (*n* = 25)	Overall improvement ≥ 50% of symptoms sustained for 4 wk after EOT: ITT (*n* = 44): 52.4% with doxepin vs. 21.7% PBO; *p* < 0.05	Yes	Doxepin: *n* = 4 (16.0%); 2 due to AEs PBO: *n* = 2 (8.0%); 0 due to AEs
Drossman DA, et al. [33]	Desipramine (*n* = 144) Initial dose: 50 mg/d for 1 wk Increase to 100 mg/d after 1 wk Increase to 150 mg/d after 2 wk PBO (*n* = 71)	Mean (SE) composite score (treatment satisfaction, global well-being, pain, health-related QOL) at wk 12 for desipramine vs. PBO: ITT (*n* = 201): 0.49 (0.02) vs. 0.45 (0.02); *p* = 0.16 PP (*n* = 153): 0.55 (0.02) vs. 0.48 (0.02); *p* = 0.03	Yes	Desipramine: *n* = 40 (29.6%); 23 due to AEs PBO: *n* = 11 (16.7%); 3 due to AEs
Talley NJ, et al. [35]	Imipramine (*n* = 18) Initial dose: 25 mg/d Increased to 50 mg/d after 2 wk PBO (*n* = 16)	Patients achieving adequate relief of IBS symptoms at last wk of tx (up to 12 wk): ITT (*n* = 34): 100% with imipramine vs. 69.2 % PBO; *p* = 0.80	Yes	Imipramine: *n* = 9 (50.0%); due to AEs NR PBO: *n* = 3 (18.8%); due to AEs NR
Vahedi H, et al. [34]	Amitriptyline (*n* = 27) 10 mg/d for 2 mo PBO (*n* = 27)	Mean total symptom score (baseline: AMI [2.5]; PBO [2.4]) At 4 wk: AMI: 1.2; *p* = 0.005 vs. baseline PBO, 1.6, *p* = 0.01 vs. baseline No significant between-group differences At 8 wk: AMI: 0.5, *p* < 0.001 vs. baseline PBO: 1.6, *p* < 0.005 vs. baseline Significant improvement with AMI vs. PBO (*p* = 0.01)	Yes	Amitriptyline: *n* = 2 (7.4%); 1 due to AEs PBO: *n* = 2 (7.4%); 1 due to AEs
Ghadir MR, et al. [42]	Doxepin (*n* = 29) Nortriptyline (*n* = 29) PBO (*n* = 29)	8 wk (*n* = 75): Abdominal pain and bloating improvement scores from baseline significantly higher with doxepin vs. nortriptyline (*p* = 0.001) or vs. PBO (*p* = 0.01); improvement in diarrhea higher with nortriptyline vs. doxepin or PBO (*p* = 0.02)	No	*n* = 12 (4 in each group; 13.8%)
Abdul-Baki H, et al. [36]	Imipramine (*n* = 59) 25 mg/d for 12 wk Optional doubling of dose at wk 2 PBO (*n* = 48)	Percentage of patients achieving global symptom relief at 12 wk: ITT (*n* = 107): 42.4% with imipramine vs. 25.0% PBO, *p* = 0.06 PP (*n* = 56): 80.6% with imipramine vs. 48.0% PBO; *p* = 0.01	Yes	Imipramine: *n* = 28 (47.5%); 14 due to AEs PBO: *n* = 23 (47.9%), 6 due to AEs
Agger JL, et al. [37]	Imipramine (*n* = 70) Initial dose: 10 mg/d for 1 wk Increasing to 25 mg/d after 1 wk Increasing to 75 mg/d after wk 2 PBO (*n* = 68)	Patient-rated overall improvement in health on CGI scale at wk 13: OR for improved outcome ITT (*n* = 125): 3.3 (95% CI, 1.6–6.8); *p* = 0.001 PP (*n* = 110): 3.8 (95% CI, 1.8–8.1); *p* = 0.001	Yes	Imipramine: *n* = 8 (11.4%); 4 due to AEs PBO: *n* = 7 (10.3%); 3 due to AEs
SSRIs				
Kuiken SD, et al. [47]	Fluoxetine (*n* = 19) 20 mg/d for 6 wk PBO (*n* = 21)	Mean (SD) threshold for pain and discomfort during rectal distension: 6 wk ITT (*n* = 40): 28 (3) mm Hg with fluoxetine vs. 29 (3) mm Hg with PBO; no significant differences	Yes	Fluoxetine: *n* = 2 (10.5%); 2 due to AEs PBO: *n* = 4 (19.0%); 4 due to AEs
Tabas G, et al. [49]	Paroxetine + HFD (*n* = 38) Initial dose: 10 mg/d EOT: 10 mg/d (23%); 20 mg/d (43%); 40 mg/d (33%) PBO + HFD (*n* = 43)	Percentage of patients with improvement in overall well-being at 12 wk 63.3% with paroxetine vs. 26.3% PBO; *p* = 0.01	Yes	Paroxetine: *n* = 8 (21.1%); 4 due to AEs PBO: *n* = 7 (16.3%); 4 due to AEs
Vahedi H, et al. [45]	Fluoxetine (*n* = 22) 20 mg/d for 12 wk PBO (*n* = 22)	Frequency of 5 abdominal symptoms (abdominal discomfort, bloating, hard stool consistency, frequency of bowel movement <3 times/wk, change in bowel habit): ITT (*n* = 44) 4 wk: all symptoms less frequent with fluoxetine vs. PBO; *p* < 0.05 for all 12 wk: differences between treatments sustained	Yes	Fluoxetine: NR; 35 AEs reported PBO: NR; 19 AEs reported
Tack J, et al. [46]	Citalopram (*n* = 11) Initial dose: 20 mg/d, first 3 wk Increased to 40 mg/d, second 3 wk PBO (*n* = 12)	Mean (SD) number of days with reduction in overall symptom severity (secondary endpoint) ITT (*n* = 23)First 3 wk: 5.7 (0.7) with citalopram vs. 7.7 (0.4) PBO; *p* < 0.05 Second 3 wk: 5.0 (0.8) with citalopram vs. 7.3 (0.5) PBO; *p* < 0.05	Yes	Citalopram: *n* = 1 (9.1%), due to AEs PBO: *n* = 1 (8.3%), due to AEs
Talley NJ, et al. [35]	Citalopram (*n* = 17) Initial dose: 20 mg/d Increased to 40 mg/d after 2 wk PBO (*n* = 16)	Patients achieving adequate relief of IBS symptoms at last wk of tx (up to 12 wk): ITT (*n* = 33): 69.2% with citalopram vs. 69.2% PBO; *p* = 0.80	Yes	Citalopram: *n* = 5 (29.4%); due to AEs NR PBO: *n* = 3 (18.8%); due to AEs NR
Masand PS, et al. [50]	Paroxetine (*n* = 36) Initial dose: 12.5 mg Increased biweekly (12.5-mg/d increments) to 50 mg/d for 12 wk PBO (*n* = 36)	ITT (*n* = 72) a wk 12: Change from baseline in composite pain score: –2.8 with paroxetine vs. –1.9 PBO; *p* = 0.82 CGI-improvement (score, 1 or 2): 69.4% (25/36) with paroxetine vs. 16.7% (6/36) PBO; *p* < 0.01 (secondary endpoint) CGI-severity (≥1-point reduction from baseline): 58.3% (21/36) with paroxetine vs. 27.8% (10/36) PBO; *p* < 0.01 (secondary endpoint)	Yes	Paroxetine *n* = 6 (16.7%); 3 due to AEs PBO: *n* = 8 (22.2%); 2 due to AEs
Ladabaum U, et al. [48]	Citalopram (*n* = 27) Initial dose: 20 mg/d for 4 wk Increased to 40 mg/d for 4 wk PBO (*n* = 27)	Self-reported weekly “adequate relief” of IBS symptoms during ≥ 3 of the previous 6 wk: ITT (*n* = 54): 44.4% (12/27) with citalopram vs. 55.6% (15/27) PBO; *p* = 0.59 OR for weekly response with citalopram vs. PBO: 0.80 (95% CI, 0.61–1.04) PP (*n* = 45): OR for weekly response with citalopram vs. PBO: OR: 0.91 (95% CI, 0.69–1.20)	Yes	Citalopram: *n* = 7 (25.9%); 7 due to AEs PBO: *n* = 2 (7.4%); 1 due to AE

^a^ Data reflect results reported in the original trial publications. ^b^ Data reported for IBS symptoms in each publication. AE: adverse event, AMI: amitriptyline, CGI: Clinical Global Impression, CI: confidence interval, EOT: end of treatment, HFD: high-fiber diet, IBS: irritable bowel syndrome, ITT: intent-to-treat, NR: not reported, OR: odds ratio, PBO: placebo, PP: per protocol, QOL: quality of life, SSRI: selective serotonin reuptake inhibitor, TCA: tricyclic antidepressant, tx: treatment.

**Table 2:** Summary of bias risk in clinical studies of antidepressants in irritable bowel syndrome.

Study	Random Sequence Generation	Allocation Concealment	Blinding of Participants	Blinding of Outcome Assessment	Incomplete Outcome Data	Selective Reporting	Rate of Discontinuations	Overall Quality
TCAs								
Heefner JD, et al. [38]	Unclear risk	Unclear risk	High risk	High risk	High risk	Unclear risk	High risk	Poor quality
Myren J, et al. [39]	Unclear risk	Unclear risk	Unclear risk	Unclear risk	*Low risk*	Unclear risk	Unclear risk	Fair quality
Nigam P, et al. [41]	Unclear risk	Unclear risk	High risk	Unclear risk	High risk	Unclear risk	Unclear risk	Poor quality
Boerner D [44]	Unclear risk	Unclear risk	High risk	Unclear risk	High risk	Unclear risk	Unclear risk	Poor quality
Bergmann ML, et al. [43]	Unclear risk	Unclear risk	Unclear risk	Unclear risk	*Low risk*	Unclear risk	Unclear risk	Fair quality
Vij JG, et al. [40]	*Low risk*	Unclear risk	High risk	*Low risk*	High risk	Unclear risk	High risk	Poor quality
Drossman DA, et al. [33]	*Low risk*	High risk	High risk	*Low risk*	High risk	Unclear risk	High risk	Poor quality
Talley NJ, et al. [35]	*Low risk*	*Low risk*	High risk	*Low risk*	High risk	Unclear risk	High risk	Poor quality
Vahedi H, et al. [34]	*Low risk*	*Low risk*	*Low risk*	*Low risk*	High risk	Unclear risk	*Low risk*	Fair quality
Abdul-Baki H, et al. [36]	*Low risk*	*Low risk*	High risk	Unclear risk	High risk	Unclear risk	High risk	Poor quality
Ghadir MR, et al. [42]	Unclear risk	High risk	Unclear risk	High risk	*Low risk*	Unclear risk	High risk	Poor quality
Agger JL, et al. [37]	*Low risk*	*Low risk*	High risk	Unclear risk	*Low risk*	*Low risk*	*Low risk*	Fair quality
SSRIs								
Kuiken SD, et al. [47]	*Low risk*	*Low risk*	*Low risk*	Unclear risk	High risk	Unclear risk	*Low risk*	Fair quality
Tabas G, et al. [49]	*Low risk*	*Low risk*	*Low risk*	*Low risk*	High risk	Unclear risk	*Low risk*	Fair quality
Vahedi H, et al. [45]	*Low risk*	*Low risk*	High risk	*Low risk*	*Low risk*	Unclear risk	*Low risk*	Fair quality
Tack J, et al. [46]	Unclear risk	Unclear risk	Unclear risk	Unclear risk	*Low risk*	Unclear risk	*Low risk*	Fair quality
Talley NJ, et al. [35]	*Low risk*	*Low risk*	*Low risk*	*Low risk*	*Low risk*	Unclear risk	*Low risk*	*Good quality*
Masand P, et al. [50]	Unclear risk	Unclear risk	*Low risk*	Unclear risk	High risk	Unclear risk	*Low risk*	Fair quality
Ladabaum U, et al. [48]	Unclear risk	*Low risk*	Unclear risk	*Low risk*	*Low risk*	*Low risk*	Unclear risk	Fair quality

SSRI: selective serotonin reuptake inhibitor, TCA: tricyclic antidepressant.

**Table 3:** Fragility index for clinical studies of antidepressants in irritable bowel syndrome.

Study	Fragility Index	Placebo Response Rate, % (*n*/*n*)
TCAs		
Heefner JD, et al. [38]	0	45.5 (10/22)
Myren J, et al. [39]	0	67.7 (21/31)
Nigam P, et al. [41]	2	0.0 (0/21)
Boerner D. [12,44]	0	53.7 (22/41)
Bergmann ML, et al. [23,43]	5	12.5 (2/16)
Vij JG, et al. [40]	0	20.0 (5/25)
Drossman DA, et al. [33]	0	40.9 (27/66)
Vahedi H, et al. [23,34]	0	40.7 (11/27)
Talley NJ, et al. [12,35]	0	68.8 (11/16)
Abdul-Baki H, et al. [36]	0	25.0 (12/48)
Ghadir MR, et al. [42]	5	16.7 (4/24)
Agger JL, et al. [37]	6	23.3 (14/60)
SSRIs		
Kuiken SD, et al. [47]	0	42.9 (9/21)
Tabas G, et al. [12,49]	1	21.7 (10/46)
Vahedi H, et al. [12,45]	6	13.6 (3/22)
Tack J, et al. [12,46]	1	8.3 (1/12)
Talley NJ, et al. [12,35]	0	68.8 (11/16)
Masand, et al. [50]	2	27.8 (10/36)
Ladabaum U, et al. [48]	0	55.6 (15/27)

SSRI: selective serotonin reuptake inhibitor; TCA: tricyclic antidepressant.
